# Postesophagectomy chylothorax: a review of the risk factors, diagnosis, and management

**DOI:** 10.1097/MS9.0000000000000809

**Published:** 2023-05-10

**Authors:** Mahdi Zarei, Majid Montazer, Sepehr Shakeri Bavil Oliyaei, Farid Jahanshahlou, Mohammad-Salar Hosseini

**Affiliations:** a Aging Research Institute; b Research Center for Evidence-Based Medicine; c Department of Cardiothoracic Surgery, Faculty of Medicine; d Students Research Committee; e Hematology and Oncology Research Center, Tabriz University of Medical Sciences, Tabriz, Iran

**Keywords:** chylothorax, esophageal cancer, esophagectomy, lymphatic system, surgery

## Abstract

Chylothorax is a crucial postoperative complication of esophagectomy. Characterized by the leakage of chyle and lymphatic fluid through the thoracic duct, chylothorax could result in pleural effusion, respiratory distress, shortness of breath, cardiac arrhythmia, electrolyte imbalance, and malnutrition. Postesophagectomy chylothorax is associated with high morbidity and mortality, and its diagnosis and management require prompt and accurate identification of risk factors and treatment strategies. A variety of strategies are available to treat postesophagectomy chylothorax, ranging from conservative management to pharmacological, lymphangiographic, and surgical treatments. This study reviews the physio-anatomical basis, disease presentation, diagnostic methods, risk factors, and management options for postesophageal chylothorax, filling the literature gap, and highlighting the importance of early recognition and timely intervention in improving patient outcomes.

## Introduction

Highlights

- Chylothorax is a relatively rare but fatal complication of esophagectomy surgery.
- Preoperative radiation, squamous histology, body surface area, and intraoperative fluid balance are some risk factors of post-esophagectomy chylothorax (PEC).
- PEC is categorized based on the required treatment and the daily chest tube output.
- Conservative and pharmacological methods are the first-line approach to managing PEC, before radiological and surgical intervention.
- Future studies should clarify the role of underlying risk factors and determine the efficacy of management methods.

Chylothorax is a crucial postoperative complication of esophagectomy. With an incidence of 0.4–10%, chylothorax is associated with high mortality^1,2^. Also, considering the anatomy of the thoracic duct (TD) and the physiology of the chyle collection, chylothorax could cause significant morbidity by developing pleural effusion, respiratory distress, and shortness of breath in patients^3^. Moreover, the persistence of the disease will expose the patient to malnutrition, electrolyte imbalance, immune suppression, and loss of protein, lipids, and fat-soluble vitamins^3^. As a highly invasive malignancy, esophageal cancer is one of the most important indications for performing an esophagectomy. Management of the condition varies from nutritional to surgical approaches^4^. Considering the limited information and controversial opinions regarding this condition’s characteristics and management methods, this study was performed to review and discuss the physiopathological basis, diagnostic methods, risk factors, and management options for postesophagectomy chylothorax.

## Anatomy and physiology

The TD is the largest lymphatic vessel of the body. With a length of 38–45 cm, it starts from the cisterna chyli at the level of the second lumbar vertebra, passes through the aortic hiatus and the posterior and anterior mediastinum, and terminates in either the internal jugular vein, the jugular-venous angle, or the subclavian vein^5^. Anatomical variations could be present in the aforementioned ‘standard’ pathway in up to 50% of the population^5^. The TD drains most of the lymph in the body except for the right side of the thorax, the right arm, the head, and the neck. The speed of chyle passing through the TD depends on the interval from food ingestion, fasting state, gut function, diet, drug intake, and physical activity^6^. Sixty to seventy percent of lipids from ingested foods enter the systemic circulation through the TD. The concentration of electrolytes, antibodies, and enzymes within the chyle is similar to that of plasma^7,8^.

## Etiology and risk factors

Several mechanisms can cause chylothorax, including compressive obstruction, direct involvement in infection, malignancy, a rupture from surgery, anomalies, dysfunction, and excess lymphatic fluid^9^. The etiology of chylothorax is divided into two categories: traumatic and nontraumatic. Malignancy is the most common cause in the nontraumatic category, and thoracic surgery, including esophagectomy, is the most common traumatic cause of chylothorax.

Different studies have proposed various risk factors for postesophagectomy chylothorax, including the anatomical variations of the TD, neoadjuvant therapies, and squamous histology^10–12^. Preoperative radiation to the mediastinum with local damage to the lymphatic system and delayed healing of the stump of small lymphatic vessels caused by lymphadenectomy, as well as the difficulty in separating the esophagus from the surrounding mediastinal structures in the field of radiation, increase the possibility of chylothorax^13,14^. In contrast, some recent studies have shown no increase in the incidence of chylothorax in patients receiving postoperative neoadjuvant chemoradiotherapy^15^.

Studies have observed an increased risk of chylothorax in patients with BMI less than 25 kg/m^2^ ^1^. Miao *et al.* also considered a high BMI and the subcutaneous fat layer as protective factors against TD damage during surgery. A study by Zhang *et al.* ^16^ indicates BMI less than 18.5 kg/m^2^ as an independent risk factor for chylothorax. Recent studies have also suggested the body surface area as a predictor for chylothorax^17^. On the contrary, in the retrospective study by Batool *et al.* ^11^, no significant correlation was discovered between patients’ age, sex, BMI, pathological T stage, serum albumin level, neoadjuvant therapy, and surgery duration, and their risk for developing postsurgery chylothorax.

Regarding the surgical approach, no statistical significance was discovered between the different surgical approaches, such as transhiatal, three-field, Ivor Lewis, or prophylactic TD ligation (TDL), and the likelihood of postsurgery chylothorax^1,18,19^. However, some studies have reported a higher risk of chylothorax during three-stage and Ivor Lewis surgeries. In the study of Ohkura *et al.* ^20^, the risk of postesophagectomy chylothorax was investigated among the two groups with TD preservation and TD resection. The study showed that TD removal after chemoradiotherapy and high intraoperative fluid balance results in a higher incidence of chylothorax after esophagectomy surgery. Ohkura *et al.* also reduced intraoperative fluid balance to less than 6.55 ml/kg/h and concluded that intraoperative fluid balance reduced the incidence of chylothorax. They explained that excessive postoperative fluid accumulation increased the interstitial fluid volume, and increased pressure inside the lymphatic vessels makes TD prone to leakage at the site of minor damages. However, in another study, no significant difference was reported in the incidence of postoperative complications, including pneumonia, anastomosis leakage, and chylothorax, between the TD resection and preservation groups^21^. Moreover, Ohkura *et al.* reported a greater incidence of chylothorax within higher stages, with an incidence of 6.4, 4.8, 11.1, and 18.8% for stages I, II, III, and IV, respectively. Therefore, TD seems more susceptible to damage if the patient has a higher clinical TNM stage. Considering the disparity between opinions and the lack of consensus on the risk factors of postsurgery chylothorax, more studies on this subject seem necessary.

## Signs, symptoms, and disease presentation

The first signs of postesophagectomy chylothorax usually are increased chest tube output volume and a yellow change in the color of chest tube secretions after the patient starts feeding after surgery^9^. When the volume of chylothorax is high (>500 ml/day), the onset may occur immediately, or it may start within 2–10 days after the traumatic event for those with slower accumulation. If the leak is slow, it may begin soon after the patient resumes oral intake after surgery. Chylothorax should be highly suspected if chest tube output exceeds 1000 ml/day, especially if four days or more have passed since the surgery^22,23^. Respiratory distress and dyspnea caused by the pleural effusion in a patient that has recently had an esophagectomy surgery could be a sign of postesophagectomy chylothorax^24,25^. In the physical examination, there may be a decrease in breath sounds and percussion dullness. In such cases, it is recommended to perform a chest radiography; if evidence of pleural effusion is observed, drainage, and analysis of pleural fluid are recommended.

Generally, right-sided pleural effusions result from TD injuries below the fifth thoracic vertebra, whereas left-sided effusions result from TD injuries above this level^26^. There is a possibility of pleural effusions on either side of the chest owing to any disease affecting the multiple lymphatic anastomoses and tributaries that feed the TD.

## Diagnosis

The chylothorax fluid can appear milky, as seen in the pleural effusion of cholesterol and empyema. The pH of the chylous fluid is usually between 7.40 and 7.80. White blood cells from chylothorax usually have a predominance of lymphocytes, reflecting the cellular composition of the lymph. Chyle has the same glucose, electrolyte, and protein content as plasma. The level of lactate dehydrogenase in the chylous pleural fluid is low due to the low concentration of lactate dehydrogenase in the chylous. The diagnosis is usually made by the analysis of a pleural fluid sample obtained from the chest tube. A definite diagnosis can be made if the triglyceride content of the fluid sample is higher than 110 mg/dl and the cholesterol content is less than 200 mg/dl^27^.

If a patient’s recent surgical history and clinical signs and symptoms suggest chylothorax, but the triglyceride content of the pleural fluid is less than 110 mg/dl, further analysis should be done by measuring the chylomicron content of the fluid. A positive result confirms the diagnosis of chylothorax, while a negative result means other etiologies should be considered. If lipoprotein electrophoresis is unavailable in the center, pleural fluid triglyceride measurement can be repeated after a high-fat meal. If the triglyceride concentration of the pleural fluid is less than 50 mg, it firmly rules out chylothorax. When interpreting the results, it is essential to consider a patient’s nutrition and fasting state. Consumption of methylene blue, olive oil, or milky solution 6–8 h before surgery helps to observe TD during surgery and prevent damage or facilitate an early diagnosis in case of chyle leakage^28^.

A pleural effusion of cholesterol and empyema are other differential diagnoses for patients with milky-appearing pleural fluid. When the cholesterol level is over 200 mg/dl, cholesterol effusion can be distinguished from chylothorax, while empyema can be identified by fever, chest pain, and leukocytosis as signs of infection^29^.

## Classification

The Esophagectomy Complications Consensus Group has proposed a classification of postesophagectomy chyle leaks based on response to treatment^30^. According to this classification, postesophagectomy chylothorax can be classified into three categories based on treatment. Another classification of postesophagectomy chylothorax has been proposed based on the daily volume of chest tube output^31^. Both classifications are presented in Table 1.

**Table 1:** Different types of postesophagectomy chylothorax, according to the Esophagectomy Complications Consensus Group (ECCG)

Classification	Definition
Type I	Requiring enteral dietary modification
Type II	Requiring TPN
Type III	Requiring interventional or surgical treatment
Type A	Daily chest tube output ≤1 l
Type B	Daily chest tube output >1 l

## Management

**Figure 1:**
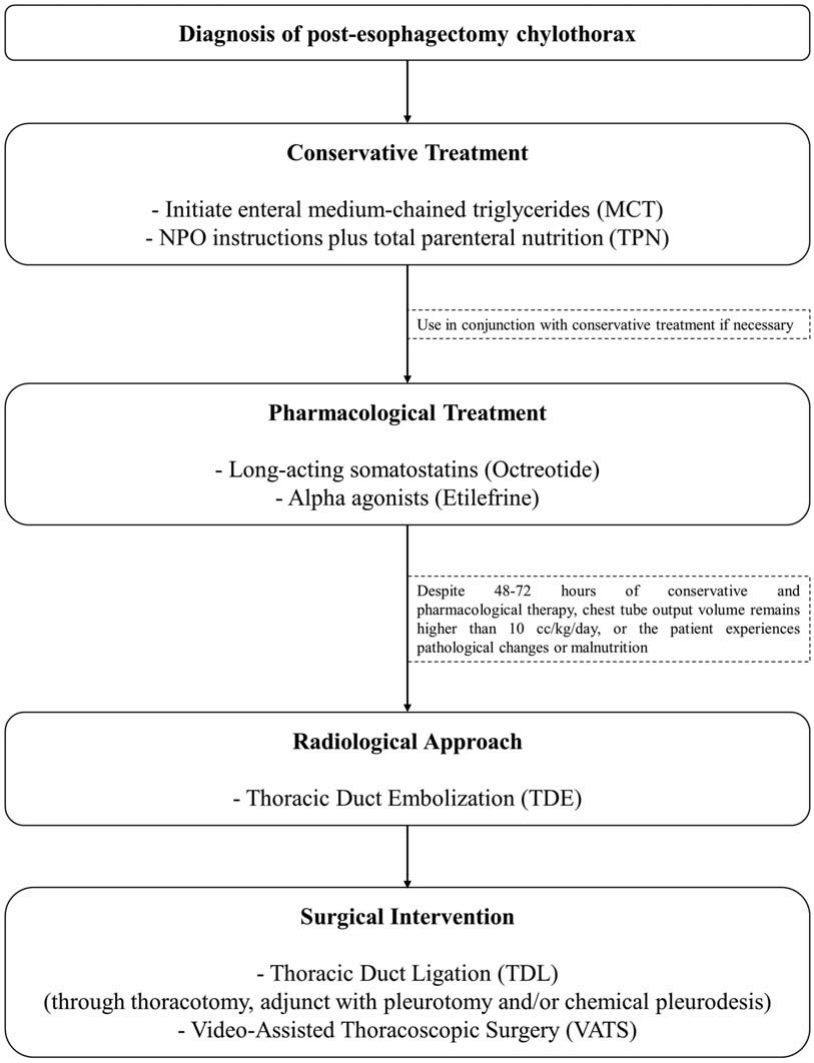
Management of postesophagectomy chylothorax.

An overview of the management of postesophagectomy chylothorax has been presented in Figure 1. The management options are discussed in the following categories:

### Conservative management

Conservative means of managing chylothorax are more effective in cases of chylothorax with chest tube output volumes of less than 10 ml/kg/day^14,32^. In such cases, on the instant of diagnosis, either enteral low-fat medium-chain triglycerides (MCT) or total parenteral nutrition (TPN) alongside Nil Per Os (NPO) instructions should be utilized to slow the chyle flow within the TD to help quicken the recovery progress^33^. It has been agreed upon that the first step in the standard management of chylothorax is either NPO (nothing by mouth) instructions plus TPN, or an enteral MCT diet^34^. If the use of MCT proves ineffective, an NPO diet and TPN can be utilized, up to 77% effective. However, the controversy has not yet been fully settled regarding the effectiveness of these two approaches. A study performed by Chalret du Rieu *et al.* ^6^ prioritizes the use of enteral MCT over TPN since MCT can be directly absorbed into the portal system, circumventing the lymphatic system and also preventing intestinal villous atrophy or changes in normal gut flora, thanks to their oral route of administration. However, it is essential to note that prolonged use of oral MCT can lead to a deficiency of linoleic acid, which is a long-chain triglyceride with systemic functions. Overall, it is recommended not to use conservative treatment for more than 2 weeks due to the possible complications of chylothorax^1^.

### Pharmacological treatment

Using long-acting somatostatins such as Octreotide and alpha-agonists such as Etilefrine is effective as adjuvant therapies for managing chylothorax^35–37^. Octreotide, with a recommended subcutaneous dose of 100 µg every 6–8 h or an intravenous dose of 50–200 µg every 8 h, leads to decreased splanchnic blood flow and, subsequently, the flow of chyle, which leads to faster recovery^38^. The sympathomimetic effect of Etilefrine (administered as an intravenous infusion of 120 mg daily for 7 days) leads to increased contraction of the TD, narrowing the lumen and decreasing flow within the TD, helping to increase the chance of recovery. The chance of recovery in patients that receive pharmacological treatments like Octreotide and Etilefrine, alongside conservative treatments such as MCT and TPN, within a 5–7 day period is reported to be as high as 80–90%^39^. Overall, varying opinions exist on pharmacological treatment for the management of chylothorax, and further studies are needed to reach a consensus on this subject.

Although there is no specific contraindication for using the pharmacological options of treatment, previous studies have shown that in patients with a chest tube drainage volume of more than 10–14 ml/kg, 3 days after the start of treatment, the probability of medical treatment failure is high^1,10^. Likewise, etilefrine should be adjusted for patients with arrhythmia or hypertension^38^.

### Lymphangiographic approach

Thoracic Duct Embolization (TDE) is a method previously used only to detect TD leaks before surgery. TDE has recently been utilized as a treatment method for chylothorax to a high degree of success^40^. Intranodal lymphangiography is performed with local anesthesia and ultrasonography guidance without general anesthesia or incisions. First, a superficial inguinal lymph node is detected, punched, and injected with an oil-based contrast agent (such as Lipidol). About an hour after the injection, adequate opacification of the cisterna chyli and abdominal and pelvic lymphatics should have been achieved. Next, the cisterna chyli is punctured, a guide wire, and microcatheter are passed through the cisterna chyli into the TD, and embolization is done proximal to the location of the leak using a glue such as N-butyl cyanoacrylate. Successful embolization of the TD is highly dependent on the technical expertise of the person performing the procedure; hence, the success rates of TDE vary between different medical facilities. Generally, a success rate of 70% is estimated, with the probability of complications arising from the procedure being estimated at 3%^40^. TDE can effectively treat postesophagectomy chylothorax with a high drain output volume. No matter how slight the chance of a successful TDE might be, it is always recommended to be tried before attempting surgical intervention for a second time since, even upon failure, it can provide valuable information regarding the patient’s anatomical TD variations and the exact location of the leak, helping with the chances of a more successful surgery. Unfortunately, as mentioned, this procedure requires proper equipment and an experienced operator to succeed, which might not be available in most medical facilities.

### Surgery

About 65% of patients undergoing an esophagectomy surgery are reoperated within 2 weeks of their first surgery^14^. This reoperation increases the chance of morbidity and mortality by 50 and 10%, respectively. This increase in morbidity and mortality rates can be attributed to the different approaches utilized for treating chylothorax, as some studies recognize reoperation within 48 h of esophagectomy as a high-risk regarding morbidity and mortality rates^41^. In contrast, other studies have shown reoperation within the same period to be a contributing factor in decreasing the number of hospitalization days for patients with an esophagectomy surgery^42^. People who develop chylothorax symptoms earlier are more likely to need surgery^1^.

A general recommendation regarding invasive management of chylothorax would be as follows: If the chest tube output remains higher than 10 ml/kg/day or the patient starts experiencing pathological changes or malnutrition, despite 48–72 h of conservative and pharmacological therapy, TDL should be considered^43,44^. Before surgical ligation, the leak’s location should be determined (and preferably, the TD should be embolized) using lymphangiography. Subsequently, if TDE fails, the surgeon should ligate or clip the TD proximal to the leak through a thoracotomy, which could be done using any or a combination of titanium clips, hem-o-lock clips, or nonabsorbable sutures^44,45^.

Mass ligation of the TD is performed at the supradiaphragmatic region of the TD between the descending aorta and the azygous vein^46^. This procedure is performed in patients with anatomical variations or unidentified ductal injury using unabsorbable sutures such as polypropylene^44^. Chemical pleurodesis using bleomycin can be used as an adjunct therapy alongside TDL since TDL will direct the flow of chyle and lymphatic fluid through collateral ducts and lymphatic-venous anastomoses.

Some studies have demonstrated the role of prophylactic TDL in reducing the risk of postesophagectomy chylothorax^47^. Meanwhile, some studies consider prophylactic TDL not to help lower the risk of chylothorax^48^. It is recommended to perform prophylactic TDL to prevent post-surgical chylothorax in patients with risk factors such as difficult mediastinal dissection.

One surgical approach to treating severe chylothorax after esophagectomy surgery is a combination of pleurectomy, TDL, and chemical pleurodesis. This approach is most effective when performed immediately after the diagnosis of chylothorax is made, and conservative and pharmacological approaches have proven to be insufficient, especially if performed within 28 days of the diagnosis of chylothorax. Du *et al.* ^28^ orally administered olive oil to the patient before surgery to help identify the leak’s location. After intubation and administration of general anesthesia, an incision is made in the appropriate intercostal space, and the parietal pleura is carefully dissected from the thoracic wall and the esophagus. Next, the TDL is performed, and finally, the space between the esophagus and the aorta above the diaphragm is sutured shut to prevent future accumulation of chyle within this space. Successful execution of this procedure has decreased the rate of sepsis, respiratory distress, and hospitalization days before the patient can be discharged. The most crucial thing about this procedure is choosing the right patient. Because if performed unnecessarily, not only does it not help increase the patient’s chance of survival, but rather imposes a high-risk surgery upon them without any justifiable benefits^49,50^.

The gold standard for the management of postesophagectomy chylothorax unlikely to respond to conservative or pharmacological methods is video-assisted thoracoscopic surgery. This method is quickly gaining popularity among physicians thanks to its lower risk of morbidity and ease of management. In this method, after intubation and administration of general anesthesia, the patient is positioned appropriately, taking into account the location of the chylothorax, and after adequately establishing the TD, thoracoscopic ligation is performed. This procedure has a reduced surgical site infection and sepsis risk and fewer hospitalization days are needed compared to thoracotomy^30^.

## Complications of long-term chyle leakage

Previous studies have shown that chylothorax is associated with an increase in the possibility of cardiac arrhythmia, pneumonia, and more extended hospitalization in postesophagectomy patients but no increase in mortality^51,52^. Considering that pneumonia is the leading cause of mortality after esophagectomy, the importance of diagnosing and treating chylothorax increases^53^. If left untreated, long-term chyle leakage can lead to a loss of protein, albumin, lipids, and vitamins and expose the patient to malnutrition^54^. It could also lead to dehydration and electrolyte imbalances, presenting with acidosis, hyponatremia, hypokalemia, and hypocalcemia^4^. Chyle also plays a vital role in the body’s immune function; therefore, long-term leaks from the TD can lead to lymphopenia (an absolute lymphocyte count less than 1500 /µl), the suppression of the immune system, and an increased risk of bacterial and viral infections^37^.

Furthermore, delayed treatment of postesophagectomy chylothorax can lead to undernutrition, pleural effusion, respiratory distress, tracheal deviation, need for intubation, tissue edema, loss of muscle mass, and the need for long-term thoracic cavity drainage. If left untreated, it could also decrease the bioavailability of drugs such as amiodarone and digoxin^53^. Therefore, it is essential to diagnose and treat postesophagectomy chyle leaks quickly and efficiently to decrease the chance of severe complications and the risk of morbidity and mortality.

## Study limitations

The current review provides a comprehensive clinical viewpoint regarding different aspects of postesophagectomy chylothorax. However, it is essential to note some limitations. This study is limited by the narrow knowledge of the topic. This review has compiled existing literature on the subject, but the number of studies on this topic is relatively small, many of which are case reports or case series, leading to limited generalizability of most findings. Moreover, information about the long-term outcomes of patients who develop chylothorax after esophagectomy is extremely limited. As such, it is unclear whether the currently proposed management strategies would improve long-term survival, which is a serious consideration, especially for cancer patients. Despite these limitations, this review provides valuable resources for clinicians treating patients undergoing esophagectomy and provides important insights into the diagnosis and management of postesophagectomy chylothorax.

## Conclusion

Chylothorax is a relatively rare but life-threatening complication of esophagectomy surgery. Considering the high-risk of morbidity and mortality and the myriad of complications that could follow postesophagectomy chylothorax, timely diagnosis and treatment of this surgical complication is vital. Generally, conservative and pharmacological methods are the first-line management before radiological and surgical intervention. However, there is still a lack of consensus regarding the management of postesophagectomy chylothorax. Future studies should more accurately determine the pertinent risk factors and the effective methods of managing postesophagectomy chylothorax.

## Ethical approval

Not applicable.

## Consent

Not applicable.

## Sources of funding

None.

## Author contribution

MZ.: conceptualization, data curation, writing - original draft; M.M.: conceptualization, data curation, supervision, writing - review and editing; S.S.B.O.: data curation, visualization, writing - original draft. F.J.: data curation, visualization, writing - original draft; M.S.H.: data curation, writing - original draft, writing - review and editing.

## Conflicts of interest disclosure

The authors declare no conflicts of interest.

## Provenance and peer review

Not commissioned, externally peer reviewed.
